# The Clinical History and Basic Science Origins of Transcutaneous Osseointegration for Amputees

**DOI:** 10.1155/2022/7960559

**Published:** 2022-03-18

**Authors:** Jason Shih Hoellwarth, Kevin Tetsworth, Muhammad Adeel Akhtar, Munjed Al Muderis

**Affiliations:** ^1^Limb Salvage and Amputee Reconstruction Service, Hospital for Special Surgery, New York, NY, USA; ^2^Department of Orthopaedic Surgery, Royal Brisbane and Women's Hospital, Butterfield St, Herston, QLD 4029, Australia; ^3^Trauma and Orthopaedic Department, Victoria Hospital, NHS Fife, Kirkcaldy, UK; ^4^Department of Orthopaedic Surgery, Macquarie University Hospital, Macquarie University, 3 Technology Pl, Sydney, NSW 2109, Australia

## Abstract

Transcutaneous osseointegration for amputees (TOFA) refers to an intramedullary metal endoprosthesis which passes transcutaneously to connect with a limb exoprosthesis. The first recognizably modern experiments and attempts occurred in the 1940s. Multiple researchers using a plethora of materials and techniques over the following 50 years identified principles and obstacles which informed the first long-term successful surgery in 1990. Unfortunately, the current mainstream TOFA literature presents almost exclusively subsequent developments, generally omitting prior research, leading to some historical mistakes being repeated. Given the increasing interest and surgical volume of TOFA, this literature review was performed to delineate TOFA's basic science and surgical origins and to integrate these early efforts within the contemporary understanding. Studying this research could protect and benefit future patients, surgeons, and implant developers as TOFA is entering a phase of increased attention and innovation. The aim of this article is to provide a focused reference of foundational research, much of which is difficult to identify and retrieve, for clinicians and researchers.

## 1. Introduction

**Figure 1:**
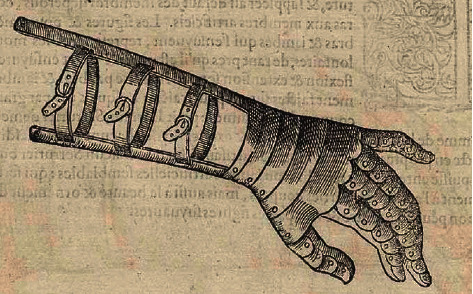
Drawing from barber/surgeon Ambroise Paré in the 1500s. The prosthetic hand features buckle straps used to suspend the hand by squeezing the residual forearm skin. Compressive suspension remains the fundamental concept in standard modern prosthetic limbs (reproduced with permission courtesy of the National Library of Medicine. Paré, Ambroise. [Les Oeuvres]. page 916. A Paris: Chez Gabriel Buon, 1585. https://www.nlm.nih.gov/exhibition/historicalanatomies/Images/1200_pixels/ixcxvi.jpg, accessed 7 Feb 2020).

The oldest known prosthesis, a great toe, is dated to 1550–700 BC Egypt [1]. The Roman general Marcus Sergius used an iron right hand and shield around 218 BC [2]. In the 1500s, Ambroise Paré fashioned prosthetic limbs resembling modern designs: a socketed extremity which attaches by squeezing residual soft tissue [3, 4] (Figure 1). Contemporary patients using traditional socket prostheses (TSP) continue to experience compression-induced skin problems and repetitive minor trauma causing intermittent prosthesis disuse and mobility dysfunction [5, 6].

**Figure 2:**
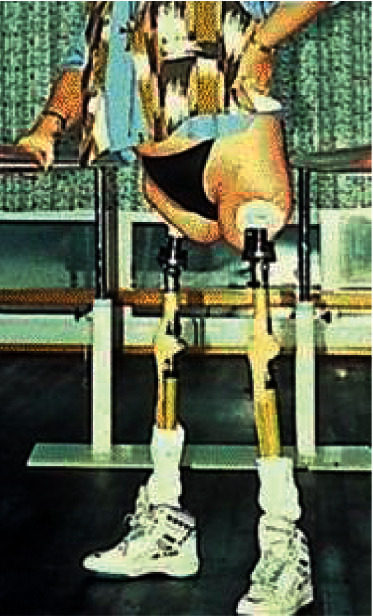
The first patient with long-term successful transcutaneous limb osseointegration. The surgery was performed on 15 May, 1990, by Rickard Brånemark in Sweden, for a young woman who lost both legs due to a street car accident. Despite her relatively short residual limbs, she is able to stand on two prosthetic legs and no compressive socket is needed. This procedure ushered in the current era of transcutaneous osseointegration for amputees (TOFA) (reproduced with permission from Li, Y., Brånemark, R. Osseointegrated prostheses for rehabilitation following amputation. Unfallchirurg 120, 285–292 (2017). https://doi.org/10.1007/s00113-017-0331-4).

On May 5, 1990, the first long-term, durable, bone-anchored prosthesis was implanted into a transfemoral amputee [7], revolutionizing amputee rehabilitation (Figure 2). Two key principles were immediately apparent. First, the bone-metal prosthesis linkage permitted nearly lossless energy transfer from person to prosthesis. Second, no longer must amputees' skin be loaded and compressed. This surgical technique is called “osseointegration,” named for the biological phenomenon. Recent reviews [8–10] have summarized implant development and clinical outcomes following TOFA since 1990. A recent editorial recognized that the preceding basic science and clinical reports are difficult to locate and generally neglected [11]. Because recent literature recognizes TOFA is safe and effective, and the United States Food and Drug Administration (FDA) has approved one implant design [12], broader use and innovation is expected. Preventing future problems by considering prior knowledge is essential [13, 14]. By providing a consolidated summary of the basic science and early clinical experiments preceding and facilitating modern TOFA, it is hoped that this article can help avoid preventable future problems.

For clarity, this article uses the word “osseointegration” to mean the biological phenomenon and the phrase “transcutaneous osseointegration for amputees” (TOFA) to mean the reconstructive surgical technique.

## 2. Methods

### 2.1. Search Strategy and Criteria

PubMed and Google Scholar were systematically searched March-October 2020 for literature before 1990 using permutations of terms such as “osseointegration,” “osseointegrated,” “osteointegration,” “osteointegrated,” “osseous integration,” and “osseous integrated.” Articles discussing TOFA designs, techniques, or basic science were selected. Clinical articles unrelated to amputees were excluded. Although the searches were performed in English, retrieved articles of any language were included and read in entirety and relevant citations retrieved whenever possible; English and German were two languages represented in this review. Journal articles, books, laboratory-based and experimental studies, conference proceedings, and abstracts were included if they fulfilled the inclusion criteria. The primary and final authors independently assessed references and manually cross-referenced potential sources. Upon completion of the review, selected additional references after 1990 were included in order to contextualize, complete, or illustrate essential principles.

## 3. Results

### 3.1. Basic Science History

#### 3.1.1. The Discovery of Titanium as a Biocompatible Material

Titanium's biocompatibility (specifically electrochemical behavior) was first investigated by Bothe in 1940 [15]. Several statements deserve quoting: “Titanium was fully as well tolerated as Vitallium and stainless steel, perhaps better in that the bone had a tendency to grow into contact with it” and “The response of bone to titanium was as good, if not better, than that to the noncorrosive alloys, in that there was more tendency for the bone to fuse with it. It possesses the advantage of being an element and hence free from the theoretical objections to alloys … If metallurgical developments of the future make it possible to work it into suitable shapes, it has the strength and hardness necessary for proper support. More experimental work is needed to prove it equal or superior to the noncorrosive alloys as a prosthetic material.” Not only were Bothe's pioneering observations fundamentally consistent with current investigations but his thoughts were impressively prophetic regarding titanium's future use as a medical material. It should be noted, however, that when interpreted from a modern perspective, the titanium Bothe used (less than 99.9% pure) would be considered an alloy and being noncorrosive (in the setting of the mammalian body as tested) could be considered appropriately grouped into the “noncorrossive alloy” category he stated.

Although contemporary literature could not be identified, Maurice Down is credited as the first to use titanium for fracture fixation, by 1947 [16, 17]. In 1951, Leventhal [18] further investigated the soft tissue and bone response to titanium, noting no evidence of soft tissue inflammation or rejection. He wrote of rabbit bone “at the end of six weeks, the screws were slightly tighter than when originally put in; at twelve weeks, the screws were more difficult to remove; and at the end of sixteen weeks, the screws were so tight that in one specimen the femur was fractured when an attempt was made to remove the screw. Microscopic examinations of the bone structure revealed no reaction to the implants. The trabeculation appeared to be perfectly normal.” He further conjectured “the use of some prostheses has not become popular because it has been felt that these would remain separate from the bone and eventually loosen. Since titanium adheres to bone, it may prove to be an ideal metal for such prostheses.” Soon after, Beder identified dogs also tolerated titanium well [19, 20]. It was only the first decade of experience with titanium, but these early researchers may have already had visions of what titanium could mean for amputee rehabilitation.

Surgical titanium research began in the 1940s because titanium was only then becoming reasonably available and affordable [18]. Although titanium is the ninth most abundant element in the Earth's crust (0.57% versus iron's 5.6% being fourth most abundant), it was only discovered in 1791 by Reverend William Gregor and 1795 by Martin Heinrich Klaproth. Elementally pure (99.9%) extraction was finally achieved in 1910 by Matthew Albert Hunter. William Kroll developed industrial production in 1946. By 1947, the United States had produced only two tons of titanium [21]. These limitations meant titanium was not widely available to any market at that time and not a familiar metal for surgeons.

**Figure 3:**
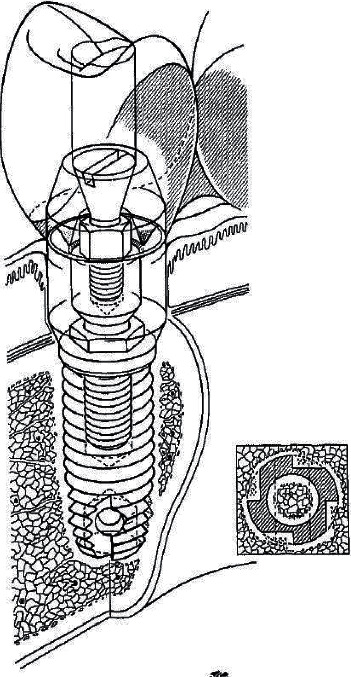
Brånemark-style dental osseointegration. Schematic shows a titanium implant with a screw fixation design. There are three components of this style of implant: 1, a titanium post that achieves osseointegration with the jaw; 2, abutment that screws into the post and remains smooth and motionless at the gingiva; and 3, the crown that is designed to match the patient's tooth (figure adapted with permission from Adell R. Lekholm U. Rockler B. R., Brånemark P. I. A 15-year study of osseointegrated implants in the treatment of the edentulous jaw. *International Journal of Oral Surgery*. 1981 Jan 1; 10(6):387–416).

But the increasing availability and its apparent biological tolerance led to its use in medical experiments. True appreciation of surgical uses of direct bone-titanium anchorage came as early as 1952 from Per-Ingvar Brånemark [22]. Independently of the aforementioned researchers, his team serendipitously also discovered that titanium screwed into rabbit bone bonded tightly; they reported no fibrous layer between implant and bone when observed by light microscopy [23] (it must be noted that this interpretation was updated as described later in this article). By 1965, following a series of canine experiments [22], he became the first to use titanium as a long-term human bone implant, specifically using it for dental implants (Figure 3) [24, 25]. By 1977, he coined “osseointegration [26, 27]” with an original meaning of the phenomenon of bone growing directly onto an implant with no intermediary fibrous tissue [28]. This definition was based on light microscopy-level histologic studies. Following studies at greater magnification revealing fibrous layers do in fact exist, the definition was modified from a histologic to a biomechanical perspective: “a process whereby clinically asymptomatic rigid fixation of alloplastic materials is achieved, and maintained, in bone during functional loading” [29]. In simpler terms, osseointegration is recognized to exist when an implant remains positionally stable in bone with chronic loading. Brånemark's discovery, research, and clinical impact on dental osseointegration is unquestionable: online searches for “Brånemark” yield hundreds of articles describing dental implants, the Brånemark System™ is a registered commercial dental implant [30], and some advocate he deserved the Nobel Prize in Medicine [31]. The 1990 implant used by his son, Rickard Brånemark, was essentially the dental implant scaled to femur size [7, 22].

Although mostly published after 1990, the contributions of Peter Thomsen at Göteborg University to the understanding of titanium-bone and titanium-skin biocompatibility are underrecognized in clinical TOFA literature and truly cannot be overstated. Serious TOFA investigators and clinicians will find most of this book [32] invaluable. Perhaps the most important discovery was that, in distinction to the aforementioned early observations by Brånemark, in successive experiments, Sennerby, Thomsen, and Ericson proved that bone does not actually grow directly onto the surface of commercially pure titanium [33]. They were the first to evaluate the bone-implant ultrastructure utilizing transmission electron microscopy which identified that collagen fibrils approach the implant surface, but a submicron thick mineral layer permanently remains between bone and titanium [34, 35]. Similar studies performed at the titanium-skin interface (stoma) identified that even clinically benign-appearing stomal skin had elevated inflammatory cell composition versus skin without a percutaneous penetration; clinically irritated skin had even greater inflammatory cell composition [36]. Additionally, whereas oral titanium implants typically have gingival epithelial attachment to the implant, skin epithelium does not form such attachments [37, 38]. This helps explain the significantly different infection rates between oral and extremity osseointegration.

#### 3.1.2. Materials Science Principles

Understanding osseointegration requires familiarity with metal materials science, particularly surface composition. Elemental and alloyed metal reacts with the surface environment, developing a “passive steady state,” also known as “passivation [39].” As examples, in atmospheric air, silver passivates (due to trace sulfur presence) to a stable tarnish (Ag_2_S), iron corrosively oxidizes to rust (Fe_2_O_3_), and titanium forms a stable oxide (TiO_2_) which remains protective *in vivo* [40]. The oxide thickness and composition depend upon the implant location (such as soft tissue, cortical bone, or marrow) and duration, though the clinical implications of this are not obvious [41]. Steel is defined as iron with carbon added, typically around 0.3–1.5% depending on desired properties such as hardness and brittleness. Corrosion occurs to iron and regular steel in the presence of water and oxygen (Fe_2_O_3_ iron oxide, rust), so stainless steel is used for orthopedic tools. Usually chrome, but sometimes nickel, is used to give steel a bulk (alloy) or surface (plated) property of “stainless,” which is a relative term indicating that corrosive iron oxidation does not occur within expected environments but instead the steel's surface passivation forms a stable protective chromium oxide (Cr_2_O_3_) or nickel oxide (NiO). Stainless steel can still undergo corrosion if subjected to repeated physical damage or excessively acidic environment [42]. Indeed, any metal will experience corrosive degradation when subjected to sufficient mechanical and acidic insult, but whereas stainless steel has proven susceptible to such deterioration in the human body [43, 44], Ti6Al4V titanium (described in the next paragraph) has proven more resilient *in vivo* [45–47].

Metal mechanical properties are also critical to understanding TOFA. Commercially pure titanium has an ultimate tensile strength of 434 MPa (CP titanium, 99.2%, grade 2), considered inadequate for adult human weight-bearing. Therefore, most medical devices alloy titanium with 6% aluminum and 4% vanadium (Ti6Al4V), improving the ultimate tensile strength to approximately 950 MPa [48]. Human cortical bone has a Young's elastic modulus between 3 and 20 GPa [49, 50], varying significantly with health, age, and direction of force (bone is anisotropic, whereas metal is effectively isotropic). The elastic modulus is approximately 110 GPa for Ti6Al4V, 190 GPa for stainless steel (316L), and 230 GPa for cobalt chrome (Co29Ch6Mo) [51, 52], the three most commonly used orthopedic implant materials. For many scenarios, a closer match of implant modulus to bone modulus is considered beneficial as stress is more uniformly transferred to the bone, improving implant longevity [53].

**Figure 4:**
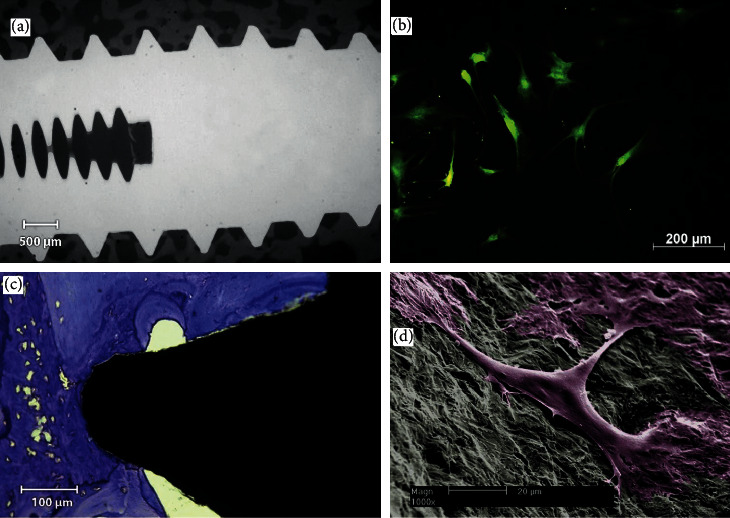
Osteocyte interaction with titanium. (a) Microradiograph with no connective tissue sheath on the implant threads after 12 weeks (magnification 20x). (b) Fluorescence microscopy images of human osteoblast cells after incubation with the 10 min eluates of a titanium implant after staining with fluorescein diacetate and propidium iodide. The presence of green (fluorescein diacetate) without red (propidium iodide) indicates living cells without dead cells. (c) After 12 weeks of healing, mature lamellar bone is evident in intimate contact with the titanium implant (toluidine blue, magnification 50x). (d) Close-up SEM images of a titanium implant seeded with human osteoblast cells. Good contact of the cells to the implant surface is shown (magnification 1000x) (reproduced from multiple figures with permission from Möller B, Terheyden H, Açil Y, Purcz N. M, Hertrampf K, Tabakov A, Behrens E, Wiltfang J, A comparison of biocompatibility and osseointegration of ceramic and titanium implants: an in vivo and in vitro study*. Int J Oral Maxillofac Surg*. 2012 May; 41(5):638–645. doi: 10.1016/j.ijom.2012.02.004. Epub 2012 Mar 8. PMID: 22406235).

The next principle is bone growth in relation to nonbiological implants. As mentioned in the preceding section, titanium and its medically used alloys do not appear to provoke an inflammatory response, leading early research using light microscopy to conclude that bone might grow directly on titanium's surface, with later electron microscopy proving this does not quite occur. A series of illustrative images are shown in Figure 4 [54]. It must be emphasized that titanium is biocompatible or perhaps better stated as bioinert; it does not elicit osteoinduction nor does it induce any recognizable upregulation or downregulation of any cell process. It remains stable within bone because it does not interfere with close interdigitation of bone growth, not because it truly unites with bone or induces special bone behavior. During titanium's formative experimental years, other materials had also demonstrated apparently stable osseointegration, such as the ceramic Cerosium (by 1963) [55, 56] and cobalt-chrome (by 1969) [57]. However, Cerosium is brittle and cobalt-chrome is substantially more dense than titanium, both relatively disadvantageous properties.

#### 3.1.3. Focused Research of Titanium's Cellular-Level Osseointegration Properties

Although researchers in the 1940s–1960s identified titanium as biologically safe, only in 1971 were the first experiments intentionally evaluating titanium's osseointegration properties published (Bothe's, Leventhal's, and Brånemark's publications were more serendipitous observations than intentional). Hirschhorn et al. [58] implanted titanium with varying surface porosity into rabbits and dogs. After 7–10 weeks, titanium-bone light photomicroscopy at up to 105x identified bone interdigitating with titanium in most samples, without intermediate tissue and without inflammatory responses. The authors felt bone tissue was very likely to be growing into the titanium, but acknowledged more definitive confirmation had to await electron microscopy. Rhinelander et al. [59] corroborated these results. In Chicago in 1971, Galante et al. [60] demonstrated rabbit and dog bone interdigitated into titanium ≥300 *μ*m by 7–10 days and achieved maximum pull-out strength of 20 kg/cm^2^ by 2 weeks. In 1972, Predecki evaluated the rate of osseointegration into titanium with channels between 95 and 1000 *μ*m. The fastest ingrowth occurred in channels with diameters between 500 and 1000 *μ*m, and greater diameter channels required more time to completely fill [61]. Stable ingrowth required at least ≥20 *μ*m of surface roughness. No bone deposited in the 95 *μ*m channel even after 18 weeks. These observations align with the structural properties of Haversian systems (osteons), Haversian canals, and osteocytes and their dendritic processes. Human lower-extremity Haversian canals are approximately 70–90 *μ*m in diameter with 246 *μ*m osteons [62]. Osteocytes are around 10 *μ*m in diameter [63] and require multiple levels of dendritic processes [64]. Channels that are too small to support an osteon are less likely to be biologically compatible.

Experiments such as these confirmed titanium to have chemical suitability (stable oxide passivation) and mechanical suitability (similar elastic modulus to bone). Optimized surface design expedited osseointegration. As manufacturing innovations allowed greater volume production, affordability improved. Titanium was becoming ready for focused clinical investigation as an osseointegrated limb replacement material, which is discussed in the next section.

### 3.2. Surgical History

#### 3.2.1. Surgical Concepts Leading to Osseointegration

**Figure 5:**
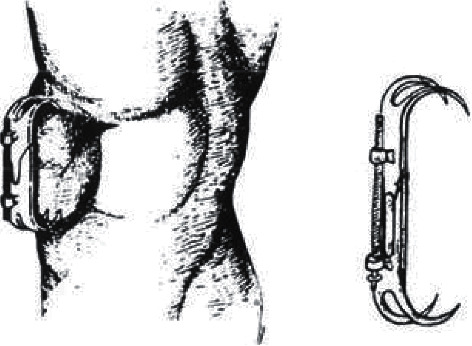
Malgaigne double-sided paired metal transcutaneous patella hook clamp (figure reproduced with permission from *Springer Nature*. Hernigou P history of external fixation for treatment of fractures. *International Orthopaedics*. 2017 Apr 1; 41(4):845–53).

Surgical innovation is generally built on a foundation of previously explored principles and discoveries, and progress with TOFA was no different. Transcutaneous orthopedic surgery did not begin with TOFA. In the 1500s, Aztec doctors were observed inserting wood into the femoral canal by Bernardino de Sahagun, an anthropologist accompanying the conquistador Hernán Cortés [65]. Although the implant did not remain transcutaneous, it was inserted through the skin. The first medical professional to document successful implementation of an orthopedic device which remained transcutaneous may have been Joseph-François Malgaigne describing his double-sided patella hook clamp. Designed in 1840, paired hooks at each end of the construct penetrated a patient's skin and clamped the superior and inferior poles of the patella, providing compression through a fracture (Figure 5). These were reported by himself in 1843 [66] and further detailed by Jules Davasse in 1846 [67]. One specific insight of Malgaigne was particularly ahead of his time: erythema, necrosis, and other signs of inflammation did not occur so long as the hooks did not slip and skin motion was eliminated. Malgaigne soon innovated an early type of external fixation [68, 69], though more recognizable external fixation instrumentation was later described by a series of still-familiar surgeons between 1850 and 1910: Philippe Rigaud, Von Heine, Bernhard von Langenbeck, Charles Bell Keetley, Clayton Parkhill, Albin Lambotte, Alessandro Codivilla, and Fritz Steinmann [68, 70–75]. In 1918, Hey Groves documented successfully treating infected femur fractures with transcutaneous intramedullary nailing, leaving the proximal portion of the nail protruding through the incision to drain [76, 77]. Such innovations proved that transcutaneous metal techniques could be safe and therapeutic.

#### 3.2.2. First Attempts at Skeletally Anchored Limb Replacements

**Figure 6:**
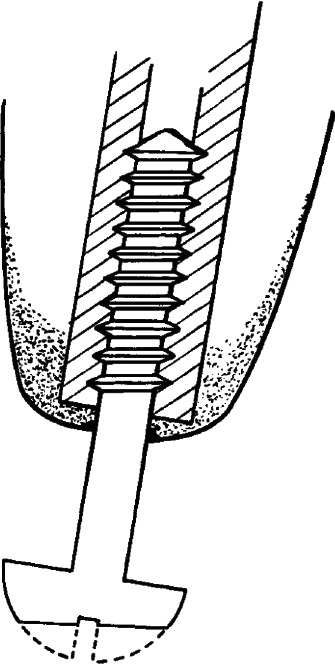
Schematic of what is likely the world's first attempt at TOFA. A Vitallium screw inserted into the intramedullary canal of dogs (figure adapted with permission from Murphy E. F. History and philosophy of attachment of prostheses to the musculoskeletal system and of passage through the skin with inert materials. *Journal of Biomedical Materials Research.* 1973 May; 7(3):275–295).

The earliest true TOFA attempts occurred in the mid-20th century history and can be traced through publications by Hulbert et al. [78] and Murphy [79], with additional historical documentation by Webster et al. [80]. The first documented skeletally linked transcutaneous prosthetic attempts were likely the pilot studies performed by Elliott Cutler and James Blodgett at Harvard University as early as 1942, sponsored by the United States Office of Scientific Research and Development. They investigated the response to stainless steel and Vitallium screws inserted into the intramedullary canal of 18 dogs (Figure 6). Vitallium retained stability better, and the researchers surmised that the implant must remain motionless relative to the bone to prevent loosening. Along with Tait Chisholm, they also implanted a Vitallium screw-style anchored tooth in a dog [79, 81]. However, by 1949, the United States Veterans Administration felt the surgical challenges for success in humans were too great and suspended further investigation.

**Figure 7:**
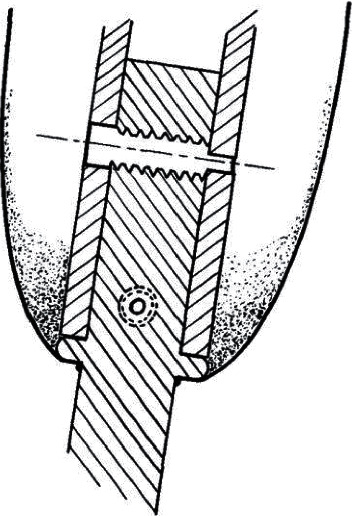
Schematic of the first skeletally implanted transcutaneous prosthetic anchor documented to be used in a human, designed by Dr. G. Dümmer in 1946. The retention mechanism was the two cross-pins through the bone and implant (reproduced with permission from Murphy E. F. History and philosophy of attachment of prostheses to the musculo‐skeletal system and of passage through the skin with inert materials. *Journal of Biomedical Materials Research*. 1973 May; 7(3):275–295).

The first attempt to replace an amputated limb with a skeletally anchored prosthesis in a human appears to have been by G. Dümmer, a general surgeon from Pinneberg, Germany, in 1946 [79]. The original source was a newspaper story, and therefore, scientific details were lacking. Dümmer treated four transtibial amputees with a stainless steel intramedullary implant which featured a cross-screw to enhance fixation and provide stability (Figure 7). These implants were removed after an apparently short period of time, possibly due to infection. Dümmer's design may not fit the strict definition of TOFA, since implant stability was provided by transverse screws rather than bone-implant interdigitation, and further details and information are no longer available. Nonetheless, this was an attempt to directly couple the skeleton to a limb prosthesis without a socket or other skin interface.

With renewed interest from the United States Veterans Administration (VA), John Esslinger began a series of experiments on dogs and a monkey between 1956 and 1969 aimed at evaluating how to overcome two challenges: (1) a stable and healthy skin-implant interface and (2) a reliable, stable integration of implant to skeleton. He experimented with stainless steel, titanium, Teflon®, and rubber implants, preferring a two-stage technique. The first stage was to insert an implant and then close the wound to allow the bone to integrate with the implant, followed by a second procedure to insert a transcutaneous connector to attach a prosthesis. Only cursory surgical technique descriptions are provided for each implant style, and although metal was in direct bone contact, it seems the aim was not truly for osseointegration to the main implant but rather to metal meshes attached to the nail-type implants. Unfortunately, he provided no figures to accompany his text. Esslinger suggested that a Teflon intramedullary implant with a mushroom-shaped cap over the distal bone end prevented bone overgrowth and seemed the most successful over several years. However, all options eventually failed and had to be removed. His report was more observational than mechanistically driven and did not feature histologic descriptions, tables of results, or any figures demonstrating these novel techniques [82].

**Figure 8:**
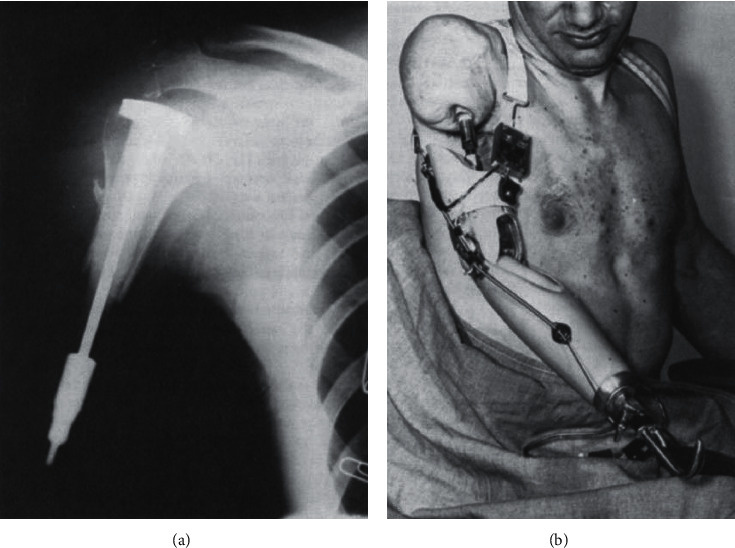
Cerosium implant used by Dr. Vert Mooney in 1967. (a) Radiograph and (b) clinical photo of a patient with a right humerus implant (figures adapted with permission from Mooney V., Predecki P. K., Renning J., Gray J. Skeletal extension of limb prosthetic attachments–Problems in tissue reaction. *Journal of Biomedical Materials Research*. 1971 Nov; 5(6):143–159).

**Figure 9:**
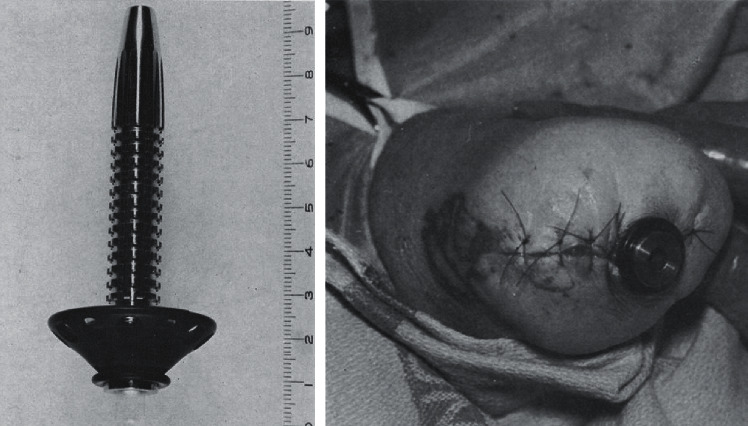
Transcutaneous skeletally linked prosthesis implant. A stainless steel implant was cemented into transhumeral amputees in the 1970s (adapted with permission from Mooney, V., Schwartz, S.A., Roth, A. M. et al. Ann Biomed Eng (1977) 5: 34. A sustained effort at limb osseointegration).

At Rancho Los Amigos, Vert Mooney reported implanting a porous ceramic rod in a patient's humerus in 1967, but within eight months, this became loose and infected (Figure 8) [83]. Although not metal, this was an attempt at TOFA, as the aim was for bone to directly attach to the implant body. One issue identified was the deeper the grooves of the implant, the longer the intraosseous vascular channels had to be to metabolically support the interdigitating bone. Recognizing the success of using polymethylmethacrylate (PMMA, bone cement) for total hip replacements [84], Mooney attempted cementing an implant into three patients. These uniformly also became loose and required removal within a year (Figure 9) [14], and Mooney appears to have abandoned further efforts at TOFA.

Also working with the VA, in 1967 [85], Charles William Hall—best known for developing the artificial heart with Michael E. DeBakey—introduced plans to achieve a clinically viable skeletally linked prosthetic leg, which they interchangeably called a “percutaneously attached artificial limb” (PAAL) or “percutaneous load-bearing skeletal extension” (PLSE). Thanks to a complete list of Hall's publications [86], his journey can be followed through approximately annual updates and is summarized below. The articles are absolutely essential reading for anyone considering future osseointegration implant development or technique innovation. The initial goals were to design an implant which would (1) be a permanent weight-bearing extension of the skeleton, (2) be able to be controlled directly by the body's tendons (artificial tendon grafts connecting host tendons to external prosthetic joints), and (3) have a natural appearance.

Concerted experimentation began in 1973 [87], mostly with Spanish goats. The goals at the start were to understand prior manufacturing inconsistencies and optimize quality control and to specifically identify mechanisms of PAAL/PLSE failure [88]. Goats seemed to have enough mobility to evade the researchers in the pasture by two months following surgery, but infection inevitably led to failure. Like others before him, Hall felt that a shock-absorbing mechanism between the implant and the bone was essential to protect the bone from the full force of impact transferred through the implant. In 1974 [89], he emphasized the need for a material to fully impede bacterial ingress (he often used nylon velour attached to the implant and sewn to the skin [89]) and again the need for a bone-implant shock regulator. Hall emphasized two critical observations early on. First was the recognition that epithelial cells (epidermis) grow until all cells achieve circumferential contact with other epithelial cells. When implants interrupt epithelial integrity, the skin tunnels deep in an attempt to attach to other epithelial cells, which marsupializes the implant. The second was that nylon velour is well-tolerated by skin epithelium; unfortunately, the permanent bond between basal cells to velour leads to the velour being migrated superficially as the basal cells mature and become the stratum corneum, eventually being completely extruded and expelled (Hall named this the “growth phenomenon”). Intramedullary implants were still unable to achieve permanent direct bond with bone, and surface treatments such as sandblasting, covering with plastic adhesive, and porous ceramic did not last beyond 7–14 months. In 1975 [90], Hall reported that the bone-implant interface was then considered the primary area of research need, since no implant (usually 5 inches long and with 0.25 inch diameter) was able to maintain successful stability. Bare metal rods were sometimes cemented, but the new philosophy was that direct bone ingrowth was preferable. Ceramics achieved bone ingrowth but were too brittle. He additionally reported [91] that animals could control the external prosthetic joint attached by artificial tendons, but these inevitably rupture. In a different article in 1975 [92], Hall emphasized that if host tissue such as skin or bone does not first occupy and continue to occupy implanted material, bacteria eventually will seed the implant and lead to infection. More than a decade later, Gristina popularized this principle as a host tissue versus bacterial “race to the implant [93].” For TOFA, the implication is that bone must quickly and permanently occupy the implant surface to prevent eventual infection. In 1976 [87], Hall summarized the prior experience and proposed an implant that was both intramedullary and extramedullary (the cortex would fit between two layers of implant). He later [94] described this as an “involuted bucket.” The main aim was to change the skin forces, hoping to avoid the “growth phenomenon.” Further “involuted bucket” details were provided in 1977 [95]. In 1978 [96], he theorized that the reaming and intramedullary location of the implants could be the main cause of the failure. Intramedullary nailing for fracture care was not yet universally familiar to orthopedic surgeons, and damage to the endosteal blood supply was often blamed for complications [97, 98]. Updated clinical experience has proven that appropriate reaming techniques do not inhibit osseointegration [8]. Implants of polished stainless steel and Proplast®, a Teflon®-based material, were used. Proplast® soon was recognized to not promote bone growth, instead resulting in fibrous tissue formation [99]. New tests of the skin interface were done in 1979 [100] using a Steinmann pin covered in Proplast®, Dacron® velour, or polished stainless steel. In a longer 1979 [101] article, he discussed the struggles of bonding skin-interface materials to implants using adhesive agents including silicones, epoxies, and acrylic adhesives. He stated the main processes leading to failure were water/water vapor damage, electrochemical reactions, and microbial or enzymatic degradation. They continued experimenting with multimaterial implants (metal structural implant with other polymer surface contact material to allow a seal against the outside). Since pull-out seemed to occur after around 12 months, they introduced implant designs which were fluted and flared wider at the proximal portion to prevent migration.

**Figure 10:**
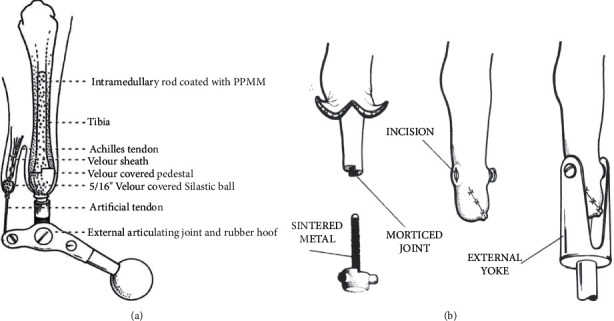
Notable designs by Charles William Hall. (a) Sketch of a 1975 attempt to preserve muscle-tendon control of the distal joint via a percutaneous artificial tendon. The inevitable artificial tendon rupture and issues with infection coming from the skin-bone interface led to this design's abandonment. (b) The final design proposed by the Hall team featured an intramedullary central titanium textured nail with three perpendicular lug attachments. The bone end was mortised to match the implant junction in order to prevent rotation before osseointegration occurred. The skin was closed over the triflanged lugs. These three lugs were later connected to an external prosthesis yoke. A key design element was that the transcutaneous bolts that connected the internal implant to the yoke was at right angle to the skin, which Hall believed would minimize tension on the skin with limb movement and thus minimize irritation, inflammation, and eventual infection (figures adapted from public domain articles. (a) Hall, C. W. Permanently attached artificial limbs. Bull Pros Res. 1975 BPR 10–42 23: 321–327. (b) Hall, C. W. A future prosthetic limb device. *J Rehab Res*. July 1985; BPR 10–42 22(3):99–102).

A major shift in experimental focus occurred in 1980 [102]. Hall switched the intramedullary metal from stainless steel to sintered titanium, characterized by microscopic particle-to-particle annealing instead of macroscopic melting/welding. He mentions partnering with the University of Illinois Chicago metallurgy professor William Rostoker who was simultaneously working with the orthopedic surgeons Jorge Galante of Rush University and Robert Ray of the University of Illinois Chicago to develop cementless hip arthroplasty stems [103, 104]. Metallurgical sintering had started to gain familiarity in the 1940s and was maturing in the 1980s [105]. In his penultimate PAAL/PLSE article [106], Hall extensively discussed principles and lessons learned during his career regarding how the skin responds to long-term percutaneous implants. Specifically detailed were the reaction of skin to deforming forces caused by the implant, the effect of dead skin cells causing a “wedge effect” if not successfully shed, the longer success of implants penetrating skin at locations of uniaxial stress (including a diagram that looks nearly identical to current transcutaneous stoma geometry), and an innovative way to maintain a transcutaneous catheter within an abdominal ileal conduit [107]. He stated “skin tension varies over the body, and an area under uniaxial tension is more likely to have a successful implant than one with areas of biaxial tensile forces. “A [percutaneous device] placed in the center of radiating forces on the skin seems doomed to failure” and refers readers to another percutaneous device review for further details [108]. In the final publication of his career in 1985 [109], he specifically identified that the aforementioned Dümmer and Mooney were the only surgeons to trial permanently attached percutaneous prosthetic limbs in humans. Hall continued to believe that such implants must be mechanically capable of weight-bearing and also fully seal the opening in the skin. He reported personal communication familiarity with a transcutaneous implant which achieved clinical success without sealing the skin, but laments that in his experience “except for [the skin continuity issue], the percutaneous load-bearing skeletal extension would now be an acceptable clinical reality.” Although titanium had already demonstrated bony stability, he was not confident the torsional stability would be adequate without a mortised distal notch cut to prevent rotation. Their best outcomes were with the triflanged intramedullary nail that has skin adherent to the flanges.  Figure 10 shows an early model sketch that was eventually abandoned, along with his final implant. He maintained that the skin interface must be resolved before human attempts were acceptable. He ended with “it is predictable that implanting a PLSE will someday become a standard orthopedic implant procedure, thereby alleviating some of the difficulties encountered by today's amputees.”

While Hall was trying to design a successful permanently attached limb prosthesis, dental osseointegration by contrast was quickly achieving excellent results. Somewhat ironically, just five years after Hall's final article and two years before his death, Rickard Brånemark implanted the first skeletally anchored prosthetic limb that achieved long-term success [7]. That implant was made of Ti6Al4V, the same material suggested by Hall and used in the world's most common current TOFA implant (Osseointegrated Prosthetic Limb, OPL). Rickard Brånemark's first patient had her contralateral femur osseointegrated one year later, and ultimately experienced over twenty years of enhanced mobility following these landmark surgeries. Although the long-term goal among most researchers is still to innovate a way to fully seal the body from the outside world at the skin level, Hall's insistence on this aspect may not be as mandatory as he believed. Based on the current implant designs that strive to minimize skin adhesion by having a polished metal-ceramic surface at the skin interface, perhaps such a seal is not necessary, or may even be detrimental, to achieving excellent clinical outcomes. Perhaps a modified Hall quotation best summarizes the current situation: “*although* the skin continuity issues are not completely understood, the percutaneous load-bearing skeletal extension *has become* a clinical reality.”

### 3.3. Future Innovations

Currently, durable bone-titanium anchorage is routinely successful, and even neural interface technology in conjunction with TOFA has proven achievable [110], if not yet routinely available. This leaves the infection prevention issue as the remaining fundamental challenge preventing TOFA from being as routinely successful and useful rehabilitation aid for amputees as the total joint replacement is for arthritic patients. A protracted conjecture of where and when this may eventually occur may be best left to a scoping-type article than a historical review, but some historic efforts, achievements, and the lack thereof are meaningful. The most traditional approaches to wound care are to either completely close skin if the environment is sterile or provide adequate tissue coverage to allow eventual skin healing via secondary intention if sterility is compromised or skin edges cannot be opposed without undue tension. Successful deviation from these principles have succeeded in temporary orthopedic situations such as the aforementioned external fixation techniques or long-term devices such as vascular access shunts [111] or cochlear implants [112], the latter of which is a type of osseointegration surgery. In some consideration, bowel diversion to an abdominal ostomy may also be considered a deviation from typical skin closure principles [113].

Various efforts at sealing the skin to the implant have not proven successful in long-term situations: Hall's aforementioned velour techniques along with various direct skin-to-metal efforts, textured or porous surfaces on cobalt-chrome [114] and titanium implants [115], and very recently adhesion protein surface modifications have been designed [116]. Can constructs that fix a static device (an osseointegrated implant) with a migratory tissue (basal skin maturation patterns) not lead to the skin either pulling the implant out or experiencing microtears which lead to inflammation and infection? It will be extremely interesting if future innovations can overcome Hall's “growth phenomenon.” A different approach of managing bacterial ingress with an egress mechanism has been pondered for at least 50 years with Murphy making the analogy of a boat's propeller shaft utilizing a labyrinth seal and bilge pump. Might there be a biological mechanism to expel incoming material through TOFA stomas in similar ways that bowel stomas do? The authors of this article have pondered whether sphincter tissue could be used at the implant-skin interface and were fascinated to read that such attempts had been made by Lawerence Friedman in the 1950s at the University of Washington [79]; unfortunately, these apparently desiccated or transformed to regular skin. Dental osseointegration achieves very low infection likely largely due to the gingiva; no articles could be found describing efforts to transplant gingiva to the skin or to successfully engineer gingival tissue. Besides teeth (and tusks), other biological examples exist where relatively hard tissue penetrates skin or other soft tissue and remains infection-free: keratin-based epidermal-specialized interfaces such as fingernails and toenails, hooves, and horns also vascularized bony tissue penetration of skin interfaces such as antlers. The details of how and why these constructs do not become infected whereas penetrating foreign bodies tend to are exceptionally complex and far beyond the scope of this historical review, but nevertheless thought-provoking. How might bio-inspired or biomimetic solutions help complete the TOFA journey? Or, conversely, could altered machining techniques which modify surface topography yield favorable skin epithelium-implant interactions [117]? The orthopedic aspect of osseointegration is sufficiently reliable, and the focus of additional research likely should focus on preventing infection, which probably requires innovations from dermatology and plastic surgery aspects.

## 4. Conclusions

The quest for a skeletally anchored limb replacement for amputees has been pursued for decades, if not for centuries. The first successful implementation was performed in 1990, and its success as well as the variety of implants that have followed was made possible by the basic science, metallurgical, and clinical innovations occurring rapidly both during the preceding 50 years and the subsequent 30 years. TOFA consistently demonstrates mobility and quality-of-life benefits for the vast majority of patients, with clinical evidence increasing rapidly during the last decade. As with any exciting technology, surgeons and implant manufacturers will continue to innovate techniques and designs. Accordingly, it is imperative to minimize patient harm and avoid wasted effort by considering as much of the available knowledge as possible. It is hoped that this article, which identifies and summarizes much of the early hard-to-find foundational TOFA literature, helps researchers more efficiently and safely innovate future TOFA solutions.

## Data Availability

No new data were used to support the study.
